# A novel method for in situ TEM measurements of adhesion at the diamond–metal interface

**DOI:** 10.1038/s41598-021-89536-2

**Published:** 2021-05-21

**Authors:** P. A. Loginov, D. A. Sidorenko, A. S. Orekhov, E. A. Levashov

**Affiliations:** 1 National University of Science and Technology MISIS, Leninskiy prospect, 4, Moscow, Russia 119049; 2 Moscow Institute of Physics and Technology, Institutskiy per. 9, Dolgoprudny, Russia 141701; 3 A.V. Shubnikov Institute of Crystallography RAS, Leninskiy prospect, 59, Moscow, Russia 119333; 4 National Research Center «Kurchatov Institute», Academica Kurchatova square, 1, Moscow, Russia 123182

**Keywords:** Characterization and analytical techniques, Microscopy, Composites, Mechanical properties

## Abstract

The procedure for in situ TEM measurements of bonding strength (adhesion) between diamond and the metal matrix using a Hysitron PI 95 TEM Picoindenter holder for mechanical tests and Push-to-Pull devices was proposed. For tensile tests, dog-bone shaped lamellae 280–330 nm thick and ~ 2.5 µm long were used as objects of study. The lamellae were manufactured using the focused ion beam technology from the metal–diamond interface of diamond-containing composite material with a single-phase binder made of Fe–Co–Ni alloy. The experimentally determined bonding strength was 110 MPa.

## Introduction

Diamond is a unique material that exhibits a combination of superior physical, chemical, and mechanical characteristics. The key advantages of diamond involve such properties as extremely high hardness (ranging from 70 to 200 GPa according to different sources)^1–3^ and thermal conductivity (2200–2600 W/(m K))^4–6^, which is only inferior to graphene. Due to its high hardness, thermal conductivity, and low coefficient of thermal expansion, diamond is used as the key component of cutting, drilling, and grinding tools^7^ and heat sinks^8,9^. Diamond is applied as a component of a composite material with a metallic, ceramic, or organic matrix (binder) or as layer in coatings deposited on different substrates. As demonstrated by numerous publications focusing on methods to improve adhesion and searching for efficient ways to produce diamond-based metal matrix composites, adhesion of binder to diamond has always been a pressing problem^10–15^.

Diamond retention capacity depends on three forces: the van der Waals forces, mechanical interlocking, and chemical bonding. The van der Waals forces are negligibly small. Mechanical interlocking depends on mechanical properties of the material's matrix, and especially on its flexural and compressive strengths. When diamond retention capacity is ensured exclusively by mechanical interlocking, the percentage of prematurely lost diamond grains is as high as 80%^16^, resulting in significant economic losses. While mechanical strength of adhesion of binder to diamond can be estimated by measuring the mechanical properties of the binder alone, assessment of adhesion ensured by chemical bonding is a rather challenging task.

Adhesion of diamond to the metal matrix is an important parameter affecting diamond retention capacity in the working layer of the tool, as well as its general performance. When designing heat sinks (especially the copper-based ones), researchers strive for improving the adhesion of binder to diamond because even small imperfections and cavities at the interface, which are inevitable because copper does not wet diamond, may dramatically reduce thermal conductivity.

It is very difficult to measure mechanical adhesion between diamond grains and the binder, which simultaneously depends on chemical bonding and mechanical interlocking. Presently, there are no standard methods for direct measuring of binder to diamond adhesion. Therefore, the methods used today involve theoretical calculations or indirect estimation, making it possible only to perform qualitative comparison for certain parameters. Special mechanical methods for estimating adhesion of binder to diamond, such as testing the strength of the diamond–metal interface under tensile loading and determining the shear strength of binder, are not commonly used and require complicated equipment^13,17,18^. For this reason, it is impossible to systematize a huge array of data on the effect of various approaches (binder composition, diamond cladding, formation of intermediate adhesive sublayers, etc.) and doping components (Ti, Cr, W, Mo, etc.) on adhesion. Materials with diamond-like carbon (DLC) coatings are also of a great importance^19,20^. Since they are exposed to high abrasive wear and severe friction conditions, the adhesion of DLC coating to the substrate strongly affects the performance. By now many approaches have been developed to improve the adhesion of DLC coatings to a substrate^21–23^, but its quantitative comparison can only be carried out by indirect methods.

Due to the small size of diamond grains, measurements of adhesion strength require micro- and nanomechanical testing methods that consider the influence of surface defects on mechanical characteristics. The Rockwell hardness tests of DLC coatings^24–26^ and the scratch test using a diamond indenter^27,28^ are the key methods that have been mastered best. In this case, the minimal stress at which the coating is detached from the substrate is a criterion for adhesion assessment. The sand abrasion test can also be conducted for coatings^24,29^. An analysis of the surface of the working layer of the tool after cutting or grinding tests is one of the most common methods used to evaluate adhesion between the diamond and the matrix in manufacturing industry. By determining the ratio between diamond grains pulled out from the binder and grains tightly connected to it after tools had been tested under identical conditions, one can draw a qualitative conclusion regarding the adhesion between diamond and a certain binder^30,31^. However, the known methods are rough and are employed only to comparatively estimate the bonding strength within similar-type groups of samples.

In this study, we propose a direct method for measuring adhesion between the diamond and the binder, which is based on using a Jeol JEM 2100 transmission electron microscope (Jeol, Japan) equipped with a Hysitron PI 95 TEM Picoindenter holder (Bruker, USA), to conduct tensile tests of lamellae cut out of bulk samples. Due to the geometry of the tested samples, adhesion can be set equal to ultimate tensile strength.

## Results

Tensile testing is widely used in materials science. Cylinder-shaped or planar samples are applied in these tests. By analyzing the stress–strain curves, one can determine a number of important strength parameters: the proportional limit, the elasticity limit, the yield strength, and ultimate strength. In our study, the ultimate tensile strength was important as this parameter determines the strain value corresponding to the maximum stress on the stress–strain curve:

1 $$\sigma = {\text{F}}_{{{\text{max}}}} /{\text{S}}_{0} ,$$

where σ is the ultimate tensile strength, Pa; F_max_ is the maximum stress, N; and S_0_ is the cross-sectional area of the sample before the tests, m^2^.

In order to successfully use the conventional tensile testing and perform quantitative measurements of adhesion of metal binder to diamond crystal, we needed to solve three problems:

1. To prepare samples for the tests;
2. To reliably determine stress applied to the sample during the tests; and
3. To accurately measure the cross-sectional area of the analyzed sample.

**Figure 1:**
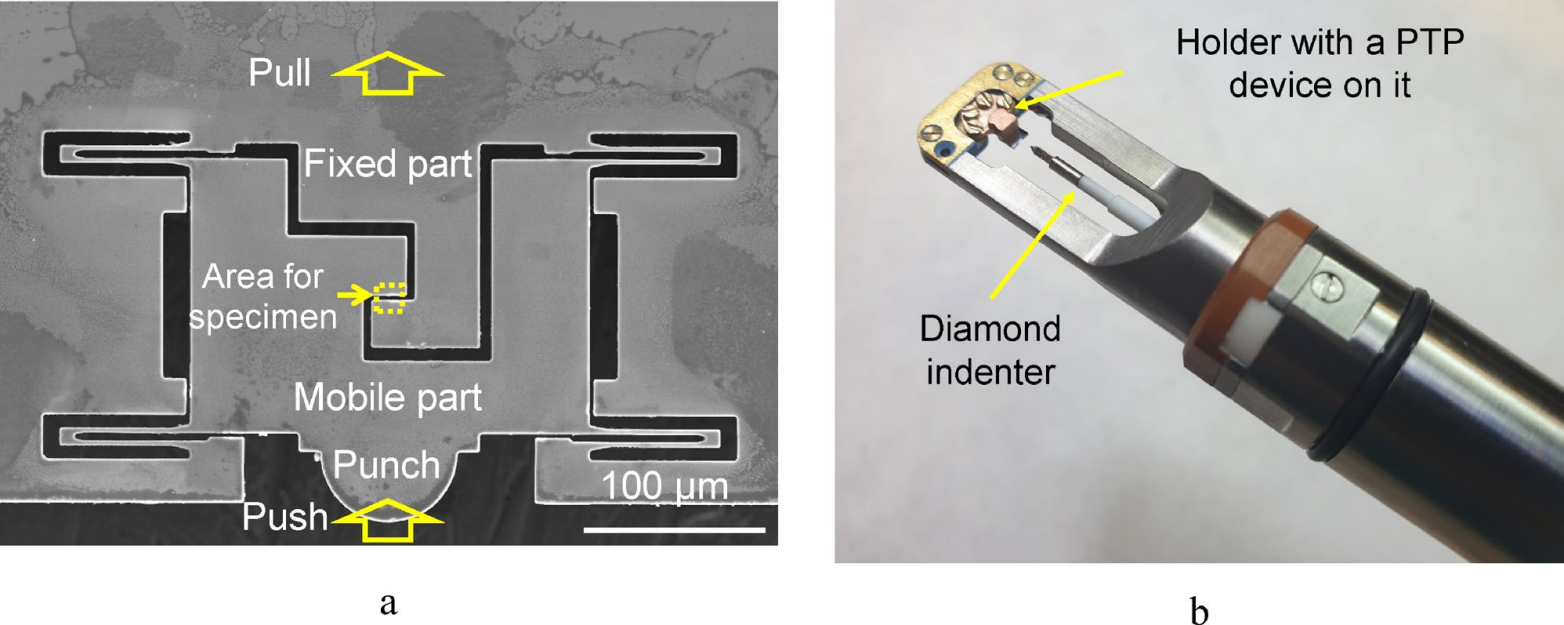
Push-to-Pull device (**a**) and the working area of the Hysitron PI 95 TEM Picoindenter holder (**b**).

It became possible to measure adhesion between the metal matrix and diamond by using microelectromechanical systems (MEMS) in situ in the TEM column. Mechanical tests were performed using Push-to-Pull (PTP) devices (Bruker, USA)^32–37^. These devices consist of a silicon spring with a slot for sample mounting and a platform (Fig. 1a) at which the diamond indenter presses (Fig. 1b). Due to the design of the PTP devices, the configuration of compressive testing can be easily transformed into that for tensile testing. During load application, the mobile part (with one of the sample sides secured on it) starts to move, thus conferring tensile stress to the sample. The tests were performed until failure of the sample.

**Figure 2:**
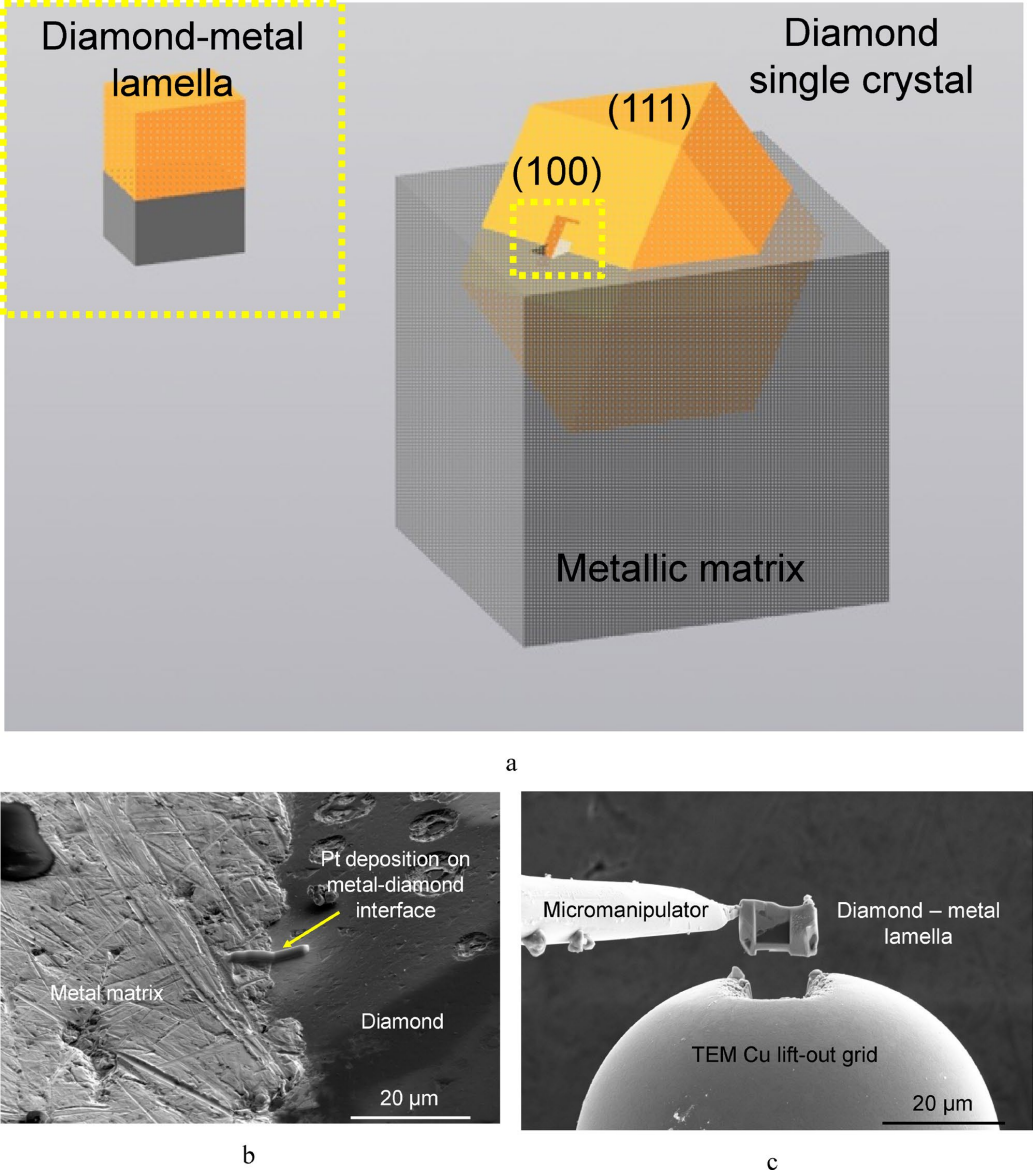

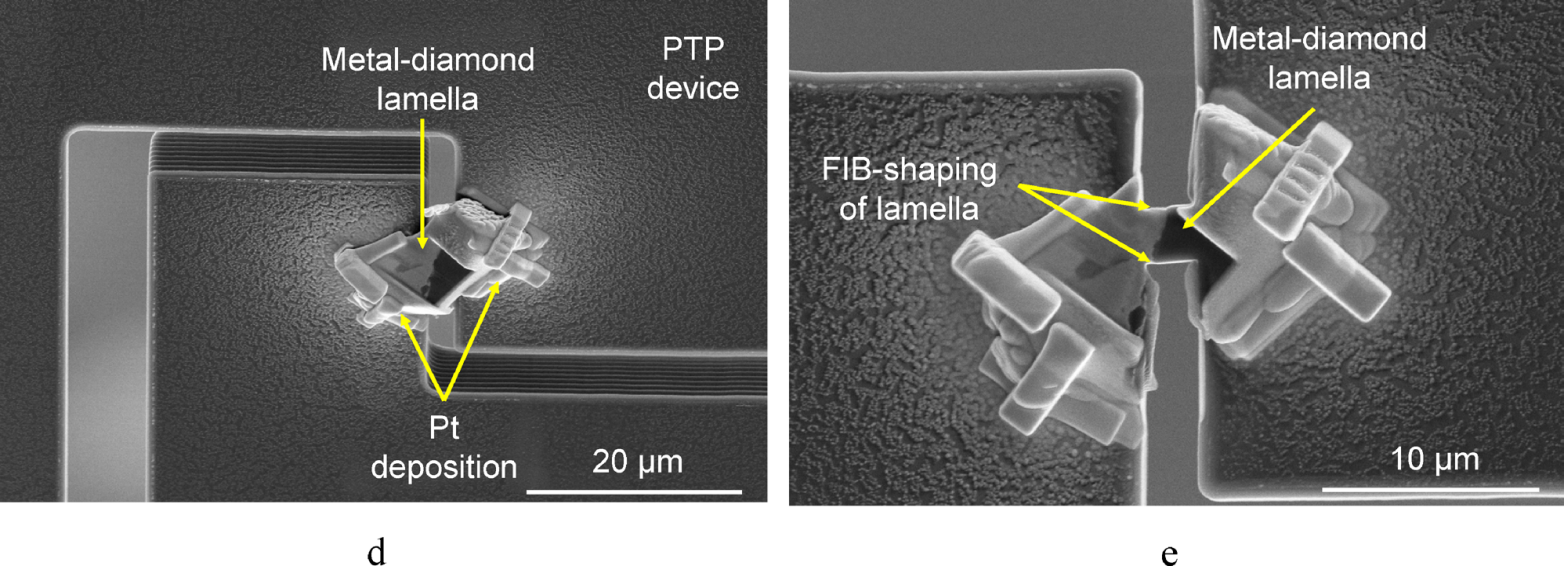
A schematic image of the original bulk metal–diamond composite (**a**) and the stages of preparing the metal–diamond lamella for the in situ tensile tests: depositing the Pt mask onto the sample surface within the interface (**b**); removing the lamellae with a micromanipulator and attaching it to the copper lift-out grid to perform additional thinning (**c**); securing the lamella to the PTP device (at a 10° tilt angle) (**d**); FIB-shaping of the lamella (**e**) (**a**—Kompas-3D v.17; https://kompas.ru/; **b**–**e**).

A composite, with its matrix consisting of the Fe–Co–Ni alloy, was the original material used to cut off the lamella for the tests^11,38^. The metal binder contained several diamond single crystals partially protruding above the surface (Fig. 2a). The lamella was cut off from the interface between the (100) face of diamond and the metal matrix using the focused ion beam technology (Fig. 2b). Ion etching was stopped when the etched layer was 8–10 µm deep. After that, the lamella was removed using a micromanipulator and mounted onto a copper lift-out grid, where it was additionally thinned to a thickness of 200–300 µm. This thickness is optimal because it ensures mechanical strength of the lamella as it is transferred from the lift-out grid to the PTP device, while on the other hand, at least half of the lamella (on the side of the diamond) will be electron-transparent for a transmission electron microscope operating at 200 kV (Fig. 2c). The lamella was oriented perpendicular to the ion source and subjected to additional thinning, so that planar parallel surfaces were obtained.

The thinned lamella was transferred to the PTP device using the micromanipulator so that the metal–diamond interface lay above the space between the supports (Fig. 2d). Next, a Pt layer was deposited onto lamella edges above the casing of the PTP device to ensure that the lamella had been securely mounted (Fig. 2e).

The load applied by the diamond indenter to the PTP device and, therefore, to the test sample, is determined using a sensor and recorded using the Hysitron Triboscan software during the tests. The high sensitivity of load measurements in the Hysitron PI 95 PicoIndenter holder is ensured due to the capacitive force/displacement transducer. This system ensures the load resolution less than 3 nN and displacement resolution less than 0.02 nm.

**Figure 3:**
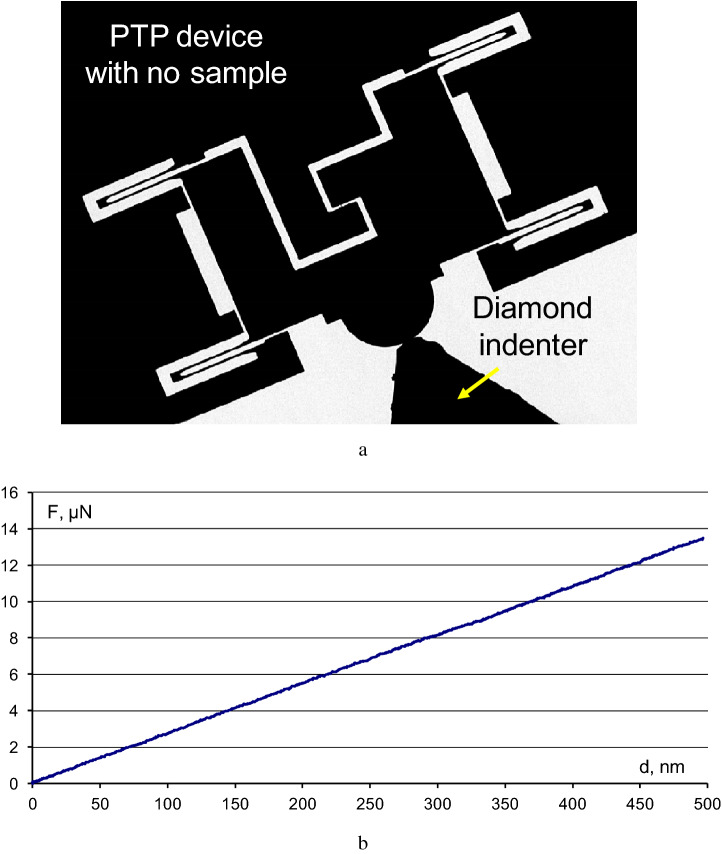
The PTP device without test sample and the indenter brought close to it (**a**) and the “zeo” curve displaying the stiffness of the PTP device (**b**).

To determine properly the maximum load applied to a sample at the moment of its failure, the intrinsic stiffness of the PTP devices should be taken into consideration. In other words, the contribution of the device should be subtracted from the resulting experimental stress–strain curve. For this purpose, the “zero” curve in a broad displacement range (up to 500 nm) was recorded prior to the main tests (Fig. 3a, Supplementary file 1). The “zero” curve had a nearly linear shape (Fig. 3b). It can be fitted using equation y = 0.027 × and subtracted from the experimental stress–strain curves using simple mathematical transformations.

**Figure 4:**
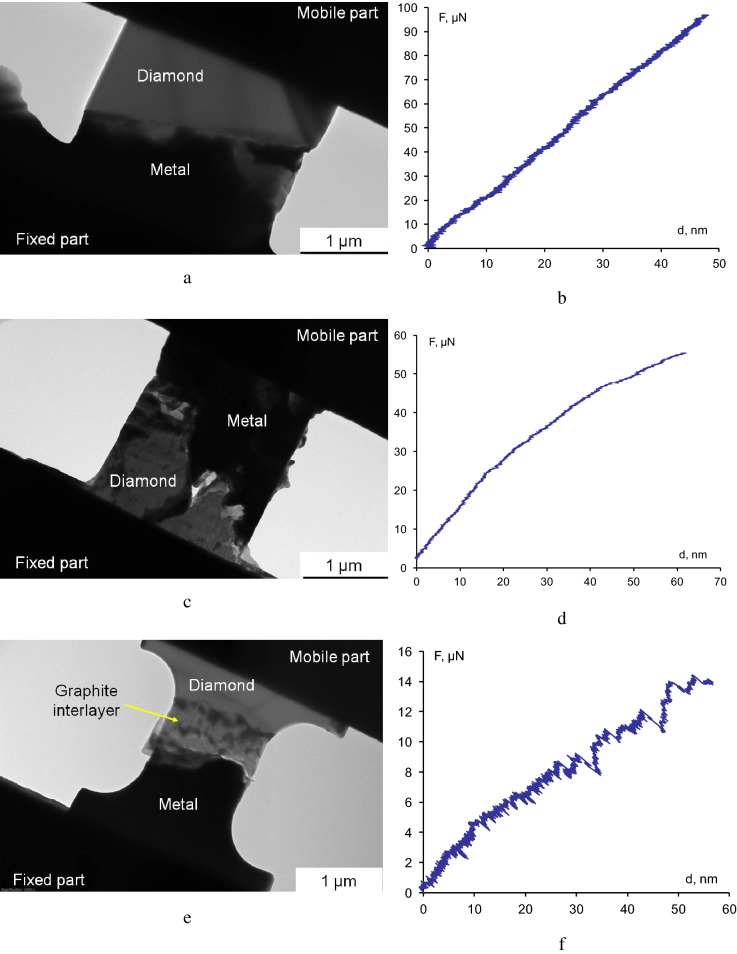
TEM images of Lamellae 1–3 before the tests (**a**,**c**,**e**) and the diagrams showing the applied load vs. indenter displacement during the tests (**b**,**d**,**f**).

To determine the adhesion, tests of the metal–diamond lamellae were run in three replicates. For the sake of convenience, let us denote them as Lamella 1 (Fig. 4a), Lamella 2 (Fig. 4c), and Lamella 3 (Fig. 4e). These lamellae differed from each other in terms of the following characteristics: cross-sectional area, thickness, and continuity of the intermediate graphite sublayer that was formed due to partial graphitization of the diamond surface during hot pressing of the original composite material (Lamella 3 had the thickest sublayer), roughness of the metal–diamond interface (the surface of the metal–diamond interface was near-planar for Lamellae 1 and 3 or complex-shaped for Lamella 2). Despite these differences, there were some common regularities in the failure behavior of the lamellae (Fig. 4b,d,f). Load-induced strain exhibited elastic behavior. The load vs. displacement curves were near-linear. Small nonlinearity during the tests of Lamellae 2 and 3 presumably results from the micro-detachment of small areas at the interface and, therefore, reduction of the contact surface area (the tests were conducted at a constant rate of indenter motion, 1 nm/s). In all the cases, the main crack in the lamellae ran near the metallic region (Supplementary file 2–4).

To determine the cross-sectional area S_0_, one needs to know the width and thickness of the Lamellae. While width can be easily measured directly on the TEM images after the camera had been calibrated with respect to the reference material, several operations need to be performed to accurately calculate thickness.

The lamella thickness was measured using the method that was first described by Kelly et al.^39^ and Allen^40^, as well as further developed by Delille et al.^41^. This method is based on analyzing the convergent beam electron diffraction (CBED). The diffraction disks in CBED patterns have the Kossel–Möllenstedt (K–M) fringes when sample thickness is greater than the extinction distance ζ_g_ (a parameter depending on structural features of the tested material). The number of fringes increases with rising thickness of the tested sample. An advantage of this method used for thickness measurements is that the CBED patterns are recorded for small regions (in our study, the convergent beam diameter was 25 nm). Hence, the lamellar thickness in the region most close to the metal–diamond interface can be measured.

**Figure 5:**
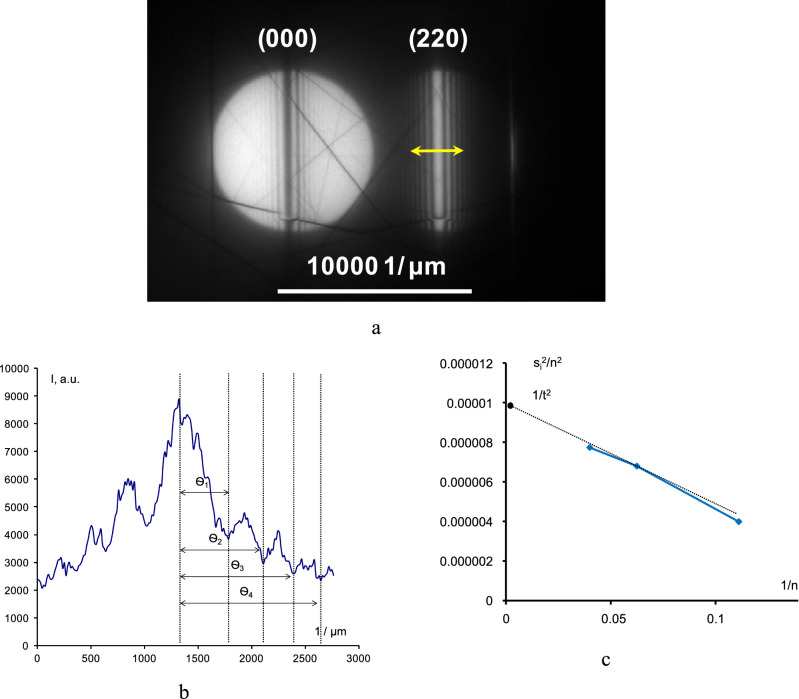
The CBED pattern of diamond area adjacent to the metal–diamond interface (**a**); the intensity profile of (220) CBED disk for the area shown with a yellow arrow (**b**); and the diagram showing (s_i_/n_i_)^2^ versus (1/n)^2^ for calculating the sample thickness t (**c**).

Figure 5a shows the diffraction disks in CBED patterns having parallel K–M fringes for Lamella 1. The sample was placed in the dual-beam position (rotated by several angles with respect to the [001] zone axis) to record a high-resolution image of the K–M fringes in the CBED pattern. The distance between the K–M fringes was determined by analyzing the light intensity profile in the (220) diffraction spot (Fig. 5b).

Thickness of the tested sample *t* is related to the ζ_g_ and s_i_ parameters (the deviation of the *i*th minimum from the primary Bragg reflection) by Eq. (2):

2 $$({\text{s}}_{{\text{i}}}^{2} + 1/\zeta_{{\text{g}}}^{2} )*{\text{t}}^{2} = {\text{n}}_{{\text{i}}}^{2}$$

where n_i_ is the consecutive number of the K–M fringe.

The following formula was used to calculate s_i_:

3 $${\text{s}}_{{\text{i}}} =\uplambda *\Delta\uptheta _{{\text{i}}} /\left( {2\uptheta _{{\text{b}}} *{\text{d}}^{2} } \right)$$

where θ_i_ is the distance between the K–M fringes; λ is the electron wavelength; θ_b_ is the angle of diffraction from the hkl plane; and d is the distance between the hkl planes.

Thickness t is determined graphically (Fig. 5c). The diagram showing (s_i_/n_i_)^2^ versus (1/n)^2^ is a straight line intersecting the (s_i_/n_i_)^2^ axis at point 1/t^2^.

The calculated thicknesses of Lamellae 1–3 are listed in Table 1.

**Table 1:** The calculated thickness, cross-sectional area, and adhesion strength (σ) of the lamellae.

Sample	F_max_ (μN)	Thickness determined by convergent beam electron diffraction (CBED) (nm)	Cross-sectional area S (m^2^)	σ (MPa)
Lamella 1	98.80	328	0.882·10^–12^	111.07
Lamella 2	55.54	324	0.612·10^–12^	90.70
Lamella 3	14.13	280	0.286·10^–12^	49.47

## Discussion

The maximum stresses were determined from the experimental dependences of stress versus displacement duing tensile testing of Lamellae 1–3 (Fig. 4b,d,f); they corresponded to 98.80, 55.54, and 14.13 µN, respectively (Table 1). Hence, the calculated ultimate tensile strengths of Lamellae 1–3 and, therefore, the values of adhesion between diamond and the metal matrix were 111.07, 90.7, and 49.47 MPa, respectively. The adhesion values for the thickest Lamellae 1 and 2 differed by 22%, thus indicating that a rather good reproducibility of the results was achieved in in situ tests. Adhesion calculated for Lamella 3 was probably somewhat underestimated. The reason is that the actual width and thickness of the interface were smaller than the dimensions of the lamella (one can see in Fig. 4 e and the Supplementary video 3 that there is a partial-thickness crack in the right-hand side of the Lamella). Most probably, Lamella 3 was insufficiently thick to ensure good mechanical strength as it was transferred from the metal–diamond composite to the PTP device.

**Figure 6:**
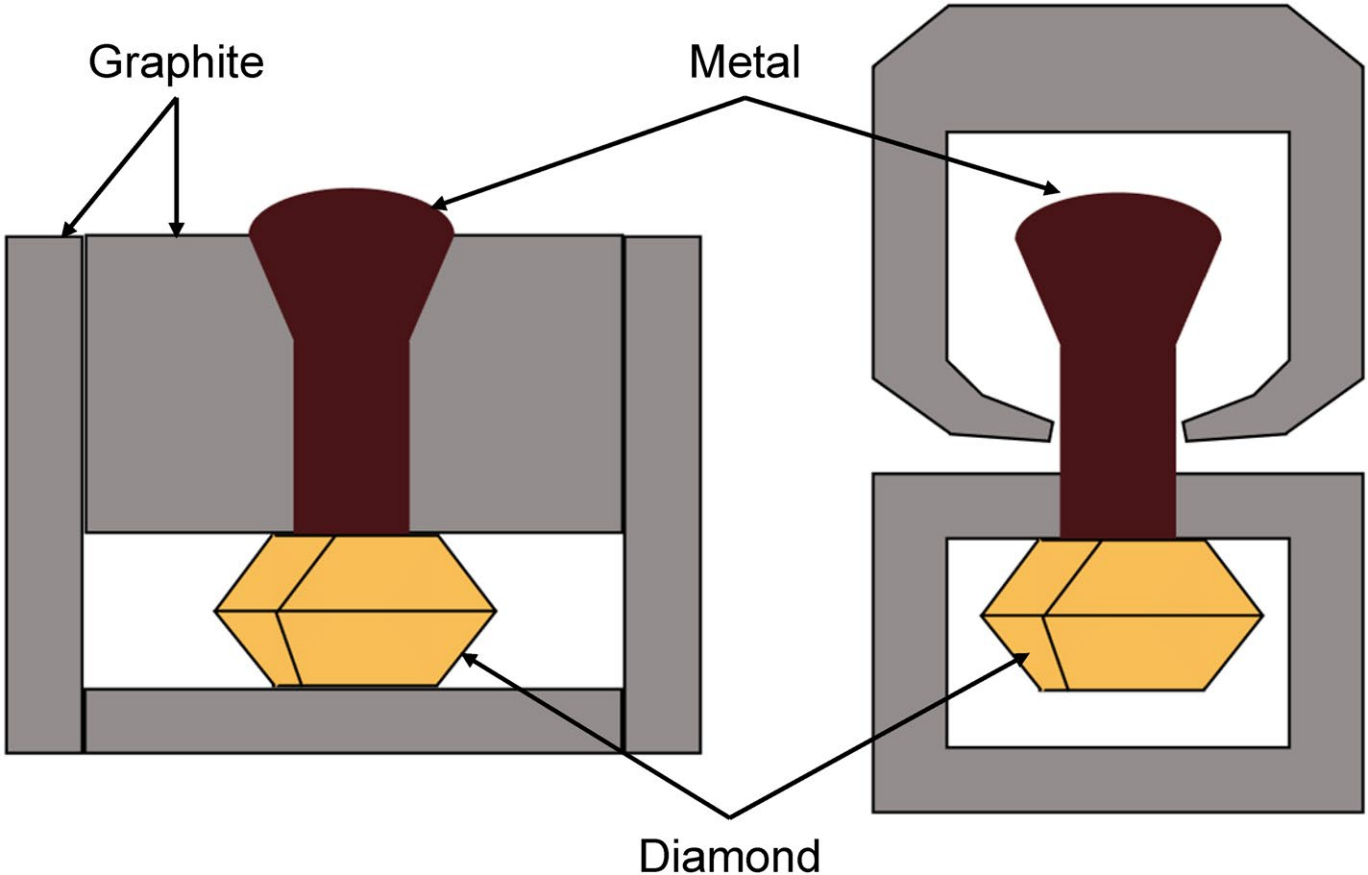
The scheme showing the deposition of the brazing metal onto diamond crystal and tensile testing of the metal–diamond interphase (adapted from^18^) (https://www.figma.com/).

As described previously, it was difficult to compare the resulting adhesion values to the literature data because of the lack of publications where adhesion would be measured using the same method. In a number of studies^18^, tensile tests were conducted as follows: the analyzed metal (alloy) in a special graphite mold was deposited onto the diamond surface (Fig. 6). After the graphite had been removed, the diamond with the hardened metal brazed onto it was placed into microgrippers of the tensile machine, and the pullout force was measured during testing. The adhesion was determined at the interface between diamond and solid WC–Co alloy doped with chromium at different concentrations. Because of the low sensitivity of this method, adhesion between the pure WC–Co alloy and diamond could not be measured due to the insufficient strength of the brazed contact. Only in the presence of a strong carbide-forming element (Cr), adhesion was sufficiently high and lay in the range of 50–180 MPa.

An important feature of the developed in situ TEM procedure for measuring adhesion is that the tests can be performed in the absence of carbide sublayers at the metal–diamond interface (i.e., in the cases when the metal matrix does not contain strong carbide-forming elements such as Cr, Mo, W, V, and Ti).

Special carefulness is required when performing sample preparation for the in situ TEM tests, and this procedure cannot be called simple. Nonetheless, the developed test configuration allows one to avoid other challenging limitations. Thus, diamond crystals not finer than 1.5 mm are required to perform the tests using procedure^18^. Crystals of this size are rarely used in industry. Therefore, it is possible to determine adhesion using procedure^18^ for the model samples only. Meanwhile, our test configuration makes it possible to determine adhesion for a fragment of the real-world sample of a cutting tool or heat sink metal–diamond composite.

Furthermore, an important advantage of the in situ TEM method is that it allows one to work with appreciably thick lamellae (> 100 nm). Sample failure occurs within the elastic strain region, where structural changes in the metal or diamond under loading do not need to be examined. Therefore, the lamellar thickness can be limited only by electron transparency of diamond (for recording the CBED patterns) and be as high as several hundred nanometers.

## Conclusions

The procedure for in situ TEM measurements of adhesion between diamond and the metal matrix by tensile testing using a Hysitron PI 95 TEM Picoindenter holder and Push-to-Pull devices has been proposed. Lamellae 280–330 nm thick and ~ 2.5 µm long with the geometry similar to that of planar samples for the conventional tensile testing were used as study objects. The lamellae were prepared from the metal–diamond interface of the diamond-containing material using the focused ion beam method.

The adhesion was determined as a ratio between the maximum stress before sample failure and cross-sectional area of the sample. For the three tested samples, adhesion lay in the range of 50–110 MPa.

## Methods

### Sample preparation

In order to fabricate a lamella with the metal–diamond interface, we prepared a compacted sample from a metal binder with composition 75 wt.% Fe–15 wt.% Co–10 wt.% Ni and several grains of single-crystal diamond (size, 40/45 mesh) preliminarily mixed into it. Sintering was conducted by hot pressing at 950 °C and pressure of 35 MPa. The powder-based binder was a single-phase alloy (α-Fe solid solution) characterized by uniform volume distribution of elements, which was manufactured using the procedure described earlier in^11,38^. The sample made of sintered diamond-containing material was subjected to mechanical grinding and polishing until diamond grains were exposed on the surface.

### Characterization methods

The method of dual-beam electron ion microscopy (on a Scios microscope, FEI, USA) was used to record the images of the compacted diamond-containing sample and the lamella, as well as to cut out the lamella and transfer it into the Push-to-Pull device. Inside the microscope, the sample was transferred from the prepared lamella to a special microchip using a micromanipulator (a 5 µm × 2 µm region was cut out from the sample by the ion beam). Since the sample was very sensitive to ion irradiation, only electron irradiation was used for transferring the samples and determining the positions where they should be fixed. Once the sample had been transferred and fixed, a “dumbbell” shape required to control sample elongation during the tensile test was cut out by etching with gallium ions using a tailored mask. As a result, the 1 µm × 0.5 µm area was selected for the experiment, where sample rupture took place.

The bright-field TEM images of the sample structure, selected area diffraction patterns (SADPs), and convergent beam electron diffraction (CBED) patterns were recorded on a Jeol JEM 2100 transmission electron microscope (Jeol, Japan) operating at 200 kV.

The images and video were recorded using an Olympus Quemesa CCD camera (Germany) at a rate of 4 frames per second.

### Mechanical testing

In situ mechanical tests were conducted using a Hysitron PI 95 Picoindenter holder (Bruker, USA) where the Push-to-Pull device with a lamella was fixed. A TI-0261 diamond indenter shaped as a truncated cone with a platform 20 µm in diameter was used to press the device punch. The course of the test was controlled using the Hysitron Triboscan software. The tests were conducted at a constant rate of indenter motion (1 nm/s) until the sample failure. The indenter was calibrated prior to each test. The maximum machine noise during the tests was no higher than 1 µN.

## Supplementary Information

Supplementary Video 1.

Supplementary Video 2.

Supplementary Video 3.

Supplementary Video 4.
